# Sleep quality and subjective well-being in healthcare students: examining the role of anxiety and depression

**DOI:** 10.3389/fpubh.2023.1281571

**Published:** 2023-12-11

**Authors:** Yihong Zhu, Runtang Meng, Chen Jiang, Nongnong Yang, Mengyi Huang, Xiaowen Wang, Wenjing Zou, Chen Lou, Ruohan Xiao, Jingjing Lu, Jiale Xu, Ulises Jiménez-Correa, Haiyan Ma, Karen Spruyt, Joseph M. Dzierzewski

**Affiliations:** ^1^School of Clinical Medicine, Hangzhou Normal University, Hangzhou, Zhejiang, China; ^2^School of Public Health, Hangzhou Normal University, Hangzhou, Zhejiang, China; ^3^Engineering Research Center of Mobile Health Management System, Ministry of Education, Hangzhou, Zhejiang, China; ^4^School of Nursing, Hangzhou Normal University, Hangzhou, Zhejiang, China; ^5^Sleep Disorders Clinic, Research Division, Medicine Faculty, National Autonomous University of Mexico (UNAM), Mexico City, Mexico; ^6^Université Paris Cité, NeuroDiderot, INSERM, Paris, France; ^7^National Sleep Foundation, Washington, DC, United States

**Keywords:** sleep quality, well-being, anxiety and depression, mediation, healthcare students

## Abstract

**Objective:**

Sleep issues, negative emotions, and health conditions are commonly co-occurring, whereas their associations among healthcare students have yet to be elucidated. This study aimed to examine whether anxiety and depression mediate the relationship between sleep quality and subjective well-being in healthcare students.

**Methods:**

A cross-sectional survey was conducted among Chinese healthcare students (*N* = 348). A battery of paper-and-pencil questionnaires—the Sleep Quality Questionnaire (SQQ), World Health Organization-Five Well-Being Index (WHO-5), and Patient Health Questionnaire-4 (PHQ-4) were applied. Descriptive analysis with means (standard deviations) and counts (proportions), Spearman correlation analysis between the SQQ, WHO-5, and PHQ-4, and mediation analysis via structural equation models were performed.

**Results:**

Correlation analysis revealed statistically significant associations between sleep quality, anxiety and depression, and well-being among healthcare students. Mediation analysis identified that poor sleep quality produced relatively low levels of self-reported well-being, which were entirely attributable to anxiety and depression.

**Conclusion:**

Sleep quality was associated with subjective well-being, and this interrelationship was fully mediated by anxiety and depression. Interventions aimed at promoting sleep quality of healthcare students may contribute to promoting their well-being by reducing anxiety and depression.

## 1 Introduction

Sleep health is an essential component of daily functioning (1). Adequate sleep quality (i.e., regularity, satisfaction, alertness, timing, efficiency, and duration) is considered integral to both physical and psychological well-being (2). Poor sleep frequently occurs in the general population due to numerous influencing factors such as high-intensity work, domestic and social responsibilities, and harmful lifestyle and behavior (3). Sleep disorders disrupt healthy homeostasis, triggering reactions in the body systems, often producing adverse health consequences (4–7). Impaired sleep is widespread in the general population (8, 9), and is associated with numerous diseases, e.g., multiple chronic conditions (10), cardiovascular disease (7, 11), impaired cognitive function (12, 13), and cancer mortality risk (14, 15). Chronic sleep issues have the potential to increase susceptibility to a variety of physical and mental illnesses, ultimately leading to a decline in overall well-being (16). Conversely, improving the qualities of sleep-onset latency, sleep dissatisfaction, and daytime sleepiness are potential avenues for enhancing physical and mental wellness (17–19). Nevertheless, the mechanism between sleep quality and well-being has yet to be comprehensively elucidated.

The burden of mental disorders is widespread across the world. Poor sleep quality and excessive daytime sleepiness can increase negative emotions, further resulting in anxiety and depressive symptoms. Sleep difficulty is a significant predictor of anxiety and depression (20). Sleep difficulty has been shown to reduce the quality of life among patients with cardiovascular diseases (21), Parkinson’s disease (22), multiple sclerosis (23), and chronic anorectal disorder (24). A meta-analysis revealed that the prevalence of depression was higher in people complaining of sleep difficulties than in the general population. Meanwhile, in people without sleep disturbances, the prevalence of depression was much lower (25). Sleep and negative emotions are interrelated, namely, dysfunction of sleep-awakening regulatory neural circuits may lead to altered emotional responses (26). Unsatisfying sleep has a known impact on raising the occurrence of psychological disturbances (27, 28); and unsurprisingly, higher levels of anxiety and depression possibly lead to a reduction in well-being (29). A previous study confirmed that anxiety and depression can negatively influence multiple elements of subjective well-being (e.g., physical well-being) (29). A longitudinal study found that anxiety disorders help predict optimal well-being over the next 10 years (30).

Poor sleep and consequent decline in well-being are common among university students, particularly those studying in healthcare professions (31–33). Data consistently reveal that healthcare students worldwide report symptoms of poor-quality sleep, and their sleep problems are more intense than non-healthcare students (34). Healthcare students’ attitudes, behaviors and lifestyles, academic pressure, and internet addiction can potentially contribute to sleep disturbances, while some variables are probably interrelated (34). Consequently, inadequate sleep has a detrimental effect on daytime functioning with prolonged drowsiness throughout the day, lowering academic performance, escalating unpleasant emotions, and risk of suicidal behavior (35, 36). Nearly one-third of healthcare students are exposed to persistent and severe anxiety or depression during the academic years due to the intensive curriculum schedule and transition from school to society (35–39). It is crucial to identify the health outcomes and underlying mechanisms between poor sleep health and negative well-being (39). Despite prior studies that have indicated the role of anxiety and depression on the sleep and well-being association, no existing evidence directly addresses the pathways underlying sleep quality and well-being in healthcare students.

A better understanding of the pathways through which sleep quality links to well-being may help to formulate tailor-made psychotherapeutic behavioral interventions. Considering the possible relationship between sleep quality, anxiety and depression, and well-being, we hypothesized significant associations among the three. Simultaneously, we constructed a mediation model and hypothesized that: (i) better sleep would be positively associated with better well-being among Chinese healthcare students; and (ii) anxiety and depression would mediate the relationship between sleep quality and well-being.

## 2 Methods

### 2.1 Study design and procedure

A cross-sectional study using anonymous and self-administered questionnaires was conducted in a healthcare student cohort. All data collection occurred in the last quarter of 2022. The study was approved by the Institutional Review Board of the School of Public Health, Hangzhou Normal University (Reference No. 20190076). The STrengthening the Reporting of OBservational studies in Epidemiology (STROBE) guidelines were followed (40).

Participants were enrolled via a stratified random sampling method. Students were not retained if they were unwilling to participate, unable to understand Chinese, or not attending school on the day. As the minimum required sample size is 15 participants for each variable, a total of at least 285 subjects were needed for this study. The final sample size of 348 exceeds this requirement. The study purpose and privacy instructions were conveyed by trained researchers prior to the survey. Each respondent acknowledged their rights and was entitled to withdraw at any point. Subsequently, a battery of paper-and-pencil surveys were administered to assess information on sleep quality, well-being, anxiety and depression, and basic socio-demographic characteristics.

### 2.2 Measures

#### 2.2.1 Sleep Quality Questionnaire (Chinese version)

The Sleep Quality Questionnaire (SQQ) is a 10-item self-report scale covering daytime sleepiness and sleep difficulty, which are two dimensions of sleep quality (41). Participants respond on a five-point Likert scale ranging from 0 (strongly disagree) to 4 (strongly agree) referring to the past month’s sleep. The total score is calculated by summing item scores with a higher score indicating poorer sleep quality. The adequate reliability, validity, and measurement invariance were well-documented by prior multi-site studies (41–45). In this study, Cronbach’s α of the SQQ was 0.811.

#### 2.2.2 World Health Organization-five well-being index (Chinese version)

The World Health Organization-Five Well-Being Index (WHO-5) is a widely used short and generic global rating scale to estimate subjective psychological well-being during the last two weeks (46). A six-point Likert scale was used, ranging from 0 to 5 (never, sometimes, less than half the time, more than half the time, most of the time, and all the time), with lower scores indicating poorer subjective physical and mental health. The questionnaire has been in existence for nearly 30 years, has been translated into over 30 languages, and has demonstrated stable psychometric properties in worldwide applications (47, 48). In this study, Cronbach’s α of the WHO-5 was 0.908.

#### 2.2.3 Patient Health Questionnaire-4 (Chinese version)

The Patient Health Questionnaire-4 (PHQ-4)^1^ is a brief and freely available instrument rated on a four-point Likert scale ranging from 0 (not at all) to 3 (nearly every day), with a higher score means more serious depressive and anxiety symptoms. The scale has a stable two-factor structure—anxiety and depression—and good psychometric properties (49, 50). In this study, Cronbach’s α of the PHQ-4 was 0.821.

#### 2.2.4 Sociodemographic

Participants completed a series of demographic questions: gender, age, home location, single-child status, family income, part-time status, leisure time sports involvement, and engagement in hobbies.

### 2.3 Statistical analysis

EpiData (version 3.1) and R (version 4.1.2) were used for data organization and data analysis. Given that researchers quickly checked the questionnaires at the time of collection and requested respondents to fill in missed questions, there were no missing values in this dataset. The “*MVN* v.5.9” package was used for multivariate normality (51). The “*dplyr* v.1.0.10” package was used for descriptive analysis: means and standard deviations (SD) were used to summarize the continuous variables while counts and proportions were used to summarize the categorical variables in descriptive statistics (52). Since the scale scores were ordinal categorical variables and the data was not normally distributed, the “*Hmisc* v.5.1–0” package was used for Spearman correlation to investigate the relationship among sleep quality, well-being, anxiety and depression (53).

The “*lavaan* v.0.6–9” (54) and “*semTools* v.0.5–5” (55) packages were used for structural equation modeling (SEM) to further explore whether anxiety and depression may explain any observed associations between sleep quality and well-being. If the direct effects of sleep quality on well-being were significant, the mediating variables were added to further calculate the indirect effects and total effects. A 1000 bias-corrected bootstrap procedure with the percentile method was evaluated for the significance of the mediation effect. The goodness-of-fit indices contain goodness-of-fit index (GFI), Tucker–Lewis index (TLI), comparative fit index (CFI), adjusted goodness-of-fit index (AGFI), parsimony normed fit index (PNFI), root mean square error of approximation (RMSEA), and standardized root mean square residual (SRMR) were reported, in which GFI, TLI, CFI, and AGFI greater than 0.90 or 0.95, RMSEA and SRMR lower than 0.08 or 0.05 for a reasonable and close fit, respectively. PNFI greater than or equal to 0.50 is considered acceptable (53, 56, 57).

## 3 Results

### 3.1 Characteristics of participants

Among the 348 valid participants (Clinical Medicine: 154; Preventive Medicine: 194), 130 (37.36%) were males (Table 1). There were slightly fewer participants younger than 20 years old (*N* = 155, 44.54%) than those greater than or equal to 20 years old (*N* = 193, 55.46%). Those who were involved in sports (*N* = 260, 74.71%) and those who had hobbies (*N* = 88, 25.28%) accounted for about three quarters and one quarter, respectively. The Kolmogorov–Smirnov test showed that the *p*-value for the SQQ was 0.026, and the PHQ-4 and WHO-5 were all less than 0.001. The *t*-test and analysis of variance (ANOVA) results were as follows: (i) worse sleep quality in the older subgroup (*p* = 0.035); (ii) more severe anxiety and depression and lower well-being were found in the group who did not participate in sports (*p_PHQ-4_* = 0.007; *p_WHO-5_* = 0.012) and who did not engage in hobbies (*p_PHQ-4_* = 0.026; *p_WHO-5_* = 0.007); (iii) healthcare students who ignored problems as the preferred coping strategies exhibited poorer sleep quality (*p* = 0.022), well-being (*p* < 0.001), anxiety and depression (*p* < 0.001) status.

**Table 1:** Characteristics of participants (*N* = 348).

Variable	*N* (%)	SQQ		PHQ-4		WHO-5	
		Mean (SD)	*t/F*	Mean (SD)	*t/F*	Mean (SD)	*t/F*
**Gender**			0.107		0.928		0.253
Male	130 (37.356)	18.862 (6.218)		3.477 (2.062)		14.508 (4.325)	
Female	218 (62.644)	18.725 (6.647)		3.729 (2.269)		14.234 (4.477)	
**Age**			4.049*		2.189		2.476
< 20	155 (44.540)	17.961 (6.224)		3.432 (2.165)		14.819 (4.345)	
≥ 20	193 (55.460)	19.430 (6.624)		3.798 (2.209)		13.948 (4.446)	
**Home location**			0.040		0.272		0.536
Urban	159 (45.690)	18.899 (6.770)		3.616 (2.308)		14.635 (4.505)	
Rural	102 (29.310)	18.608 (6.323)		3.735 (2.277)		14.147 (4.615)	
Suburban	87 (25.000)	18.747 (6.186)		3.552 (1.879)		14.011 (4.010)	
**Single-child status**			0.121		0.187		0.001
Single-child	129 (37.069)	18.915 (6.578)		3.581 (2.068)		14.271 (4.200)	
Non-single-child	219 (62.931)	18.694 (6.438)		3.667 (2.269)		14.374 (4.548)	
**Family income**			0.848		0.508		0.632
< 10,000 CNY	145 (41.667)	18.924 (6.429)		3.779 (2.367)		13.959 (4.649)	
≥ 10,000 CNY	203 (58.333)	18.670 (6.532)		3.532 (2.062)		14.606 (4.234)	
Part-time status			1.932		0.420		0.065
Do part-time job	33 (9.483)	20.303 (6.664)		3.879 (1.883)		14.091 (4.340)	
No part-time job	315 (90.517)	18.616 (6.452)		3.610 (2.225)		14.362 (4.430)	
**Leisure time sports involvement**			0.032		7.502*		7.991**
Yes	260 (74.713)	18.708 (6.658)		3.450 (2.087)		14.681 (4.366)	
No	88 (25.287)	18.977 (5.962)		4.182 (2.414)		13.318 (4.432)	
**Engagement in hobbies**			0.586		4.864*		6.506*
Yes	249 (71.552)	18.594 (6.637)		3.470 (2.203)		14.735 (4.478)	
No	99 (28.448)	19.232 (6.081)		4.051 (2.126)		13.333 (4.111)	
**Preferred coping strategies**			3.847*		10.224***		7.326***
Active copies	191 (54.885)	18.738 (5.981)		3.634 (2.075)		14.639 (4.024)	
Push through	124 (35.632)	18.089 (6.835)		3.242 (2.050)		14.613 (4.702)	
Ignore problems	33 (9.483)	21.576 (7.323)		5.121 (2.759)		11.545 (4.637)	

Bold fonts stand for different variables. The unit of the Monthly household income is CNY (1 CNY ≈ 0.140 US dollars). SQQ, Sleep Quality Questionnaire; PHQ-4, Patient Health Questionnaire-4; WHO-5, World Health Organization-Five Well-Being Index; SD, standard deviation; *p < 0.050; **p < 0.010; ***p < 0.001.

### 3.2 Correlations among sleep quality, well-being, and anxiety and depression

The Spearman correlation was performed among the subscale scores and total scores of the SQQ, PHQ-4, and WHO-5. As shown in Table 2, there were statistically significant correlations (*p* < 0.001) between all scores, ranging from 0.339 to 0.924. Due to the WHO-5 scoring, it correlated negatively with other scores clustered around −0.409 and −0.696. The association between the total scores of the SQQ and PHQ-4 was 0.514, while the total score of the WHO-5 was significantly, and negatively, related to the SQQ (*r* = −0.526). Similarly, the total score of the WHO-5 was negatively correlated with the PHQ-4 (*r* = −0.696).

**Table 2:** Correlations of the primary variables in the study for participants (*N* = 348).

Measures	SDS	DSS	SQQ	Anxiety	Depression	PHQ-4
DSS	0.381					
SQQ	0.686	0.924				
Anxiety	0.339	0.422	0.464			
Depression	0.362	0.409	0.462	0.622		
PHQ-4	0.383	0.461	0.514	0.891	0.897	
WHO-5	−0.409	−0.468	−0.526	−0.604	−0.655	−0.696

Color gradient represents correlation level. Red represents a positive correlation. Blue represents a negative correlation. SDS, Sleep Difficulty Subscale; DSS, Daytime Sleepiness Subscale; SQQ, Sleep Quality Questionnaire; PHQ-4, Patient Health Questionnaire-4; WHO-5, World Health Organization-Five Well-Being Index.

### 3.3 The mediating role of anxiety and depression

**Figure 1:**
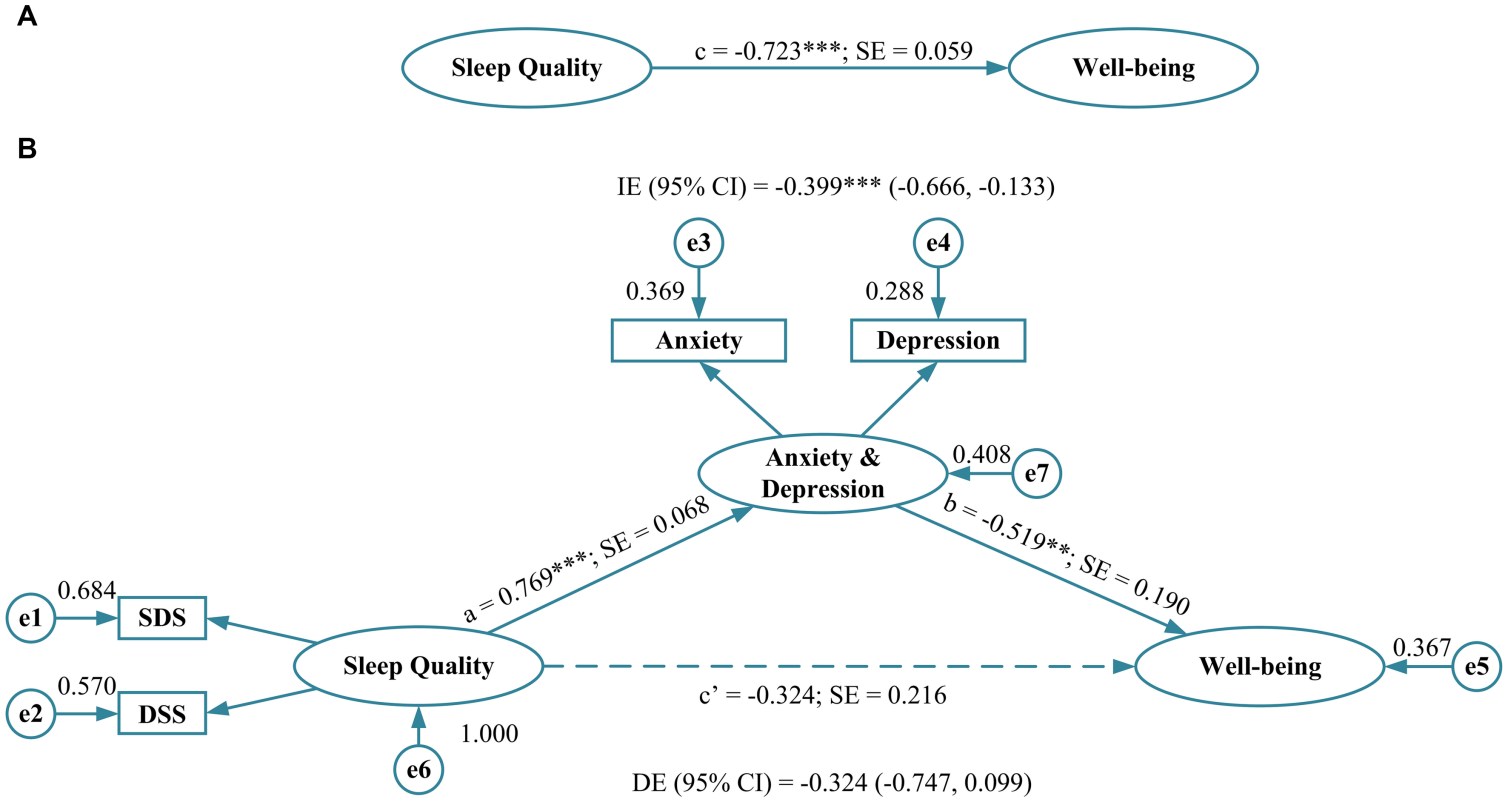
Path coefficients for simple mediation analysis on sleep quality. **(A)** The direct effect of sleep quality on well-being and **(B)** the effect of sleep quality on well-being when anxiety and depression is included as a mediator. a, b, c, and c’: standardized regression coefficients; dotted line represents the effect of sleep quality on health condition when anxiety and depression is included as a mediator. SDS, Sleep Difficulty Subscale; DSS, Daytime Sleepiness Subscale; SE, standard error; IE, indirect effect; DE, direct effect; **p < 0.010; ***p < 0.001.

The total effect (*c* = −0.723, *p* < 0.001, standard error [SE] = 0.059) of sleep quality on well-being was first estimated (Figure 1A), which revealed a significant association and resulted in the conditions necessary for the mediator model construction. Subsequently, anxiety and depression were integrated into the model as a mediator. The standardized regression coefficients from sleep quality to anxiety and depression (*a* = 0.769, *p* < 0.001, SE = 0.068) and from anxiety and depression to well-being (*b* = −0.519, *p* < 0.010, SE = 0.190) were both significant, while the direct effect (*r* = 0.324, *p* > 0.050, 95% CI: −0.747, 0.099) from sleep quality to well-being was not significant. Simultaneously, the indirect effect (*r* = −0.399, *p* < 0.001, 95% CI: −0.666, −0.133) of sleep quality on well-being through anxiety and depression was statistically significant, implying that anxiety and depression function as full mediators between sleep quality and well-being. The model showed excellent goodness-of-fit indices (RMSEA = 0.000, SRMR = 0.010, GFI = 0.997, TLI = 1.004, CFI = 1.000, and AGFI = 0.987), except for the PNFI of 0.299 which was slightly below the threshold (Table 3).

**Table 3:** Evaluation of the goodness-of-fit of the mediation model (*N* = 348).

GOF index	Index	Threshold
Absolute measures		
RMSEA (90% CI)	0.000 (0.000, 0.082)	≤ 0.080
SRMR	0.010	≤ 0.080
GFI	0.997	≥ 0.900
*χ^2^/df*	0.777	2–3
Incremental fit measures		
TLI	1.004	≥ 0.900
CFI	1.000	≥ 0.900
Parsimony measures		
AGFI	0.987	≥ 0.900
PNFI	0.299	≥ 0.500

GOF index, goodness-of-fit index; RMSEA, root mean square error of approximation; SRMR, standardized root mean residual; GFI, goodness-of-fit index; *χ*^2^, Chi-square; df, degree of freedom; TLI, Tucker-Lewis index; CFI, comparative fit index; AGFI, adjusted goodness-of-fit index; PNFI, parsimony normed fit index; CI, confidence interval.

## 4 Discussion

The current study provided empirical support for the relationship between sleep quality and well-being among healthcare students, while examining an important potential mechanism—depression and anxiety. Our findings confirmed that sleep quality was directly related to well-being, but such a link was fully mediated by depression and anxiety. Consequently, healthcare students who had high-quality sleep might have lower levels of anxiety and depression, which was associated with better well-being. This study expanded on previous research to highlight the relationship between sleep quality and well-being in healthcare students, including important variables related to negative emotions (34, 39, 58).

Correlation analysis demonstrated that sleep quality, including sleep difficulty and daytime sleepiness, was correlated with anxiety and depression. The relationships between these variables were shown in previous studies; sleep disturbance, psychological distress, and health impairment seem to be commonly intertwined (59–61). Our findings were also congruent with the phenomenon of unsatisfactory or poor sleep being relevant to the onset of mental disorders, as well as social, interpersonal, and self-health impairment (60). Previous prospective analyses have shown insomnia to be strongly predictive of impending anxiety and depression (62). Anxiety and depression have also been found to be predictor variables of sleep-related symptoms and to serve as mechanisms underlying future insomnia in the general population (62, 63). Previous studies have proposed that people with insomnia do not have higher levels of daytime sleepiness than individuals without insomnia, but this is only on an individual basis and is based on assessing physiological indicators only (64–66). A large body of literature has suggested other mechanisms at play in insomnia. Although this study assessed sleep quality primarily through sleep difficulty and daytime sleepiness, a formal diagnosis of sleep disorders was not conducted. Previous research has found that comorbid anxiety and depression are strongly associated with somatic health problems and subjective well-being (67–69). A study based on the Chinese population reported that improving sleep quality and alleviating anxiety would be attributed to decreasing somatic symptoms (60). These findings suggest that the interrelationship between sleep quality and self-perceived health is not directional, but instead, a complicated two-way process, where sleep quality influences well-being, and vice versa.

Given the extensive adverse outcomes on individual health, we should pay more attention to depressive and anxiety problems for healthcare students who are potentially at high risk. Mediation analysis supported our hypothesis, i.e., the sleep quality and well-being connection was fully mediated by anxiety and depression. Respondents with poor sleep quality were more likely to experience anxiety and depression, which in turn influenced well-being. Our findings align with results in the literature that highlight the protective role of alleviating anxiety and depression on sleep quality and a series of health events (70–72). A three-wave longitudinal study, for instance, indicated that anxiety and depression mediate the relationship between self-reported adaptability and insomnia in university students (72). The association between exposure to adverse childhood experiences and increased somatic symptoms in adolescents could be explained through anxiety and depression symptoms (69). In perimenopausal and postmenopausal women, the reduction of anxiety and depression was found to be helpful in improving sleep quality for those who were plagued by hot flashes (70). In terms of psychological health, a cross-sectional study demonstrated that anxiety fully mediated the connection between sleep disturbance and recent suicide attempts, and that relief from anxiety might reduce the risk of suicide attempts in individuals with sleep disorders (71). These findings suggest that anxiety and depression could represent one pathway through how sleep quality connects to well-being. A deepened understanding of the association between, on one hand, sleep quality and self-reported well-being, and on the other hand, remission of anxiety and depression might provide avenues for continued efforts to promote well-being.

Enhancing the well-being of healthcare students can begin with the model’s starting point, sleep quality, and its full mediators, anxiety and depression. Although sleep is a necessity, it is underappreciated. Comprehensive education can help to increase the recognition of sleep and the potential harm of poor sleep quality among healthcare students (64, 73). A critical step regarding anxiety and depression is to raise the recognition of psychiatric disorders and reduce the stigma about seeking psychological help (37, 65). Prolonged exposure to such negative emotions without seeking help may have a negative impact on academic performance, professionalism, empathy, and so on (66, 74, 75). Developing positive thinking practice courses, stress management training, and adjusting medical education programs may be possible solutions (76–78).

### 4.1 Strengths and limitations

Previous research has chiefly concentrated on one-way or two-way interactions between sleep quality, psychologies, and well-being, but few studies have focused on the potential mediating influence of anxiety and depression among healthcare students. The current study allowed for a holistic understanding of how exposure to poor or adequate sleep quality affects outcomes for healthcare students. The association between sleep quality and well-being was found to be entirely mediated by anxiety and depression. The high risk of exposure to poor sleep in this group might result in severe anxiety and depression and therefore reduced self-perceived well-being.

There are several study limitations. Respondents were recruited from a single-center sample and might not be generalized to the general population of healthcare students across China or other countries. This study did not include students from other majors as a comparison. Another limitation was that self-report questionnaires were conducted to measure sleep quality, negative emotions, and psychosomatic well-being, which might introduce potential reporter bias. Moreover, the cross-sectional design limited the ability to determine how sleep quality changed subjective well-being through anxiety and depression. A longitudinal study, relatively, would be more appropriate for examining the variation of well-being via sleep quality and emotions. Lastly, prior psychological conditions and use of medications were not assessed, which could have potentially influenced the results.

### 4.2 Future directions

Research on large, multi-center samples is needed to provide more comprehensive evidence for capturing sleep quality in different cohorts. Longitudinal examinations should be collected to elucidate causality, while considering potential confounders that might affect sleep quality, such as seasonal effects and course changes. Simultaneously, the influence of factors such as sleeping medication, antidepressants, cigarettes, and alcohol in this model can be further explored. Best approaches to apply the findings of the study to influence public policy or conduct educational activities is an area for further exploration.

## 5 Conclusion

The present cross-sectional study makes initial advances to clarify the mechanism underlying the connection between sleep quality and subjective well-being. Poor sleep quality may result in decreased well-being in healthcare students through anxiety and depression. Conducting regular screening of sleep quality and psychological conditions, providing health education to enhance the awareness of physical and mental health, and adopting targeted interventions such as sleep health education, positive thinking training, psychological counseling, and group exercise for some high-risk groups may help to promote the well-being in healthcare students.

## Data availability statement

The data generated or analyzed during this study are not publicly available due to restrictions imposed by the ethics committee. The dataset supporting the conclusions is available upon reasonable request to the corresponding author.

## Ethics statement

The studies involving humans were approved by the Institutional Review Board of School of Public Health, Hangzhou Normal University, China. The studies were conducted in accordance with the local legislation and institutional requirements. The participants provided their written informed consent to participate in this study.

## Author contributions

YZ: Validation, Writing – original draft, Writing – review & editing. RM: Validation, Writing – original draft, Writing – review & editing, Conceptualization, Data curation, Funding acquisition, Investigation, Methodology, Project administration, Resources, Software, Supervision. CJ: Formal analysis, Methodology, Software, Validation, Visualization, Writing – original draft, Writing – review & editing. NY: Validation, Writing – original draft, Writing – review & editing. MH: Validation, Writing – review & editing. XW: Writing – review & editing. WZ: Writing – review & editing. CL: Writing – review & editing. RX: Writing – review & editing. JL: Writing – review & editing. JX: Writing – review & editing. UJ-C: Validation, Writing – review & editing. HM: Resources, Validation, Writing – review & editing. KS: Methodology, Validation, Writing – review & editing. JD: Methodology, Validation, Writing – review & editing.

## Footnotes

^1^Retrieved from: https://www.phqscreeners.com; accessed on 29 August 2019.
